# Effect of human library intervention on mental health literacy: a multigroup pretest–posttest study

**DOI:** 10.1186/s12888-022-03725-5

**Published:** 2022-01-28

**Authors:** Eva Yin-han Chung, Tasha Tin-oi Tse

**Affiliations:** 1 Department of Special Education and Counseling, The Education University of Hong Kong, 10 Lo Ping Road, Tai Po, New Territories Hong Kong; 2 Centre for Special Educational Needs and Inclusive Education, The Education University of Hong Kong, Tai Po, Hong Kong

**Keywords:** Inclusion, Psychiatry, Health literacy, Health education, Stigmatization

## Abstract

**Background:**

Mental health literacy (MHL) is an evolving concept encompassing knowledge of mental illness, help-seeking options, perceived stigma, and discrimination. This study aimed to test the effectiveness of a human library intervention at enhancing MHL. A human library intervention was adopted to enhance MHL in this study. The human library intervention aims to establish a positive framework and safe space for dialogue between readers and a ‘human book’. It works to promote dialogue, reduce prejudice, and encourage understanding of people who are regarded as disadvantaged or in a minority group.

**Methods:**

An experimental approach with a multigroup pretest–posttest design was adopted. Forty-five participants aged between 18 and 23 years were recruited and randomly assigned to the experimental group (human library intervention), comparison group (didactic teaching session), or control group (no intervention). Adapted vignette-based MHL scale scores were used as the outcome measures. The overall and subscale scores were included in the analysis.

**Results:**

The human library intervention group showed a significant improvement in overall MHL compared with the other two groups. In a multivariate analysis of the variance in subscale scores, the intervention was shown to significantly reduce stigma and preferred social distance, but had no significant effect on knowledge acquisition.

**Conclusions:**

The human library intervention is effective at enhancing overall MHL and reducing stigma and preferred social distance. Further studies are suggested to further develop the MHL construct, human library interventions, and the MHL scales for consolidating evidence-based practice.

## Highlights

- Human library intervention enhanced mental health literacy.
- Human library interventions reduced stigma and preferred social distance towards people with mental illness.
- Both human library and didactic teaching interventions were not significant in enhancing the knowledge acquisition on common mental disorders.

## Introduction

The concept of mental health literacy (MHL) has been developed for two decades. It was originally defined as ‘the knowledge and beliefs about mental disorders which aid their recognition, management or prevention’ [1]. It was then further developed and expanded to include knowledge of how to prevent mental disorders, recognition of when a disorder is developing, knowledge of help-seeking options and available treatments, knowledge of effective self-help strategies for milder problems, and first-aid skills to support others who are developing a mental disorder or are in a mental health crisis [2]. However, it mainly promotes intellectual changes that aim to improve the acquisition of knowledge about mental illness. The current conceptualisation of MHL emphasises the importance of attitudes towards people with mental illness and includes reducing stigma and discrimination against them [3]. Perceived stigma and discrimination create intra-personal barriers that affect help-seeking behaviour and intervention efficacy [4]. Improved MHL at both the individual and community levels will improve mental health outcomes, promote early identification, and increase the use of mental health services [3].

Low MHL is associated with a high degree of personal stigma, stigmatising attitudes towards mental disorders, and discrimination towards people with mental disorders [5]. This may eventually discourage people from seeking appropriate diagnostic and intervention procedures to manage their illnesses [6]. MHL is weakly understood in non-Western countries [7]. From a cultural perspective, traditional Chinese values and folk understandings of mental illnesses may be responsible for the low MHL in the Chinese population [8]. There is a strong sense of shame and negative stereotypes about the dangers associated with perceptions of mental illness in Chinese communities [9]. Previous studies have suggested that Chinese people tend to somaticise the symptoms of depression and thus suppress the emotional components of the disorder [10]. Rather than acknowledge the psychological distress associated with a mental disorder, Chinese people tend to express it as a physical symptom. As a result, somatic organ-based language is more frequently used to replace expressions of emotions and psychological problems. The level of MHL, in turn, influences peoples’ attitudes towards mental illness [8].

Commonly used intervention strategies for increasing MHL are community campaigns, propaganda, information services, educational talks and mental health first aid training [2, 11]. These strategies mostly involve a didactic approach, which aims to provide teaching and information to the target audience to improve their knowledge acquisition in recognizing mental health problems, and teach them how to offer help and support to the people with mental health problems [11]. Most mental health campaigns in Hong Kong are in the early stage of delivery and include only an educational component. They seldom incorporate contact with people who have lived experiences of mental health problems [9]. The human library was, therefore, proposed as an innovative and participatory approach to enhance MHL in this study.

The human library can be used as an innovative method to promote dialogue, reduce prejudice, and encourage understanding of people who are regarded as disadvantaged or in a minority group [12]. The human library aims to establish a positive framework and a safe space for dialogue between readers and a ‘human book’. Unlike the didactic teaching approach, it emphasises personal conversation rather than a presentation or lecture to teach knowledge on mental illness. In practice, guests with diverse backgrounds and life experiences are invited to speak to people in the human library session. Common topics discussed with such guests include cultural, religious, social, and ethnic differences. It is not just a presentation of a story, but an engagement and interaction and an exchange from a lived perspective. The human library thus provides a platform for communication between human ‘books’ and ‘readers’ with the intent to embrace diversity and equity, promote social inclusion, and reduce stigma [13].

Research evidence on the process and outcomes of human library intervention is limited. However, the impact of the human library intervention on promoting inclusion has been tested in several studies. Orosz et al. [14] tested the effectiveness of a human library intervention at reducing prejudice towards Roma and lesbian, gay, bisexual, and transgender (LGBT) people. Their results suggested that prejudice towards Roma and LBGT people reduced significantly as a consequence of the human library intervention. Another study examined the effectiveness of a human library intervention at reducing social distance from Roma, Muslim, dark-skinned, and transgender people and homonegativity [15]. Groyecka et al. (2019) found that participating in the human library significantly changed the preferred social distance from Muslims, but did not significantly change homonegativity. These two studies preliminarily confirmed the effectiveness of human library intervention at reducing prejudice. However, both of these experimental studies used only experimental groups and lacked a control or comparison group in their research design, which may decrease the validity of the results.

This study aimed to test the effectiveness of a human library intervention at enhancing MHL. The objectives of the study were as follows:

1. to test the effectiveness of the human library intervention at increasing overall MHL among young people in Hong Kong;
2. to test the effectiveness of the human library intervention at improving the knowledge of mental illness among young people in Hong Kong;
3. to test the effectiveness of the human library intervention at reducing stigmatisation of mental illness among young people in Hong Kong; and
4. to test the effectiveness of the human library intervention at reducing the preferred social distance from people with mental illness among young people in Hong Kong, as compared to a didactic educational approach and a control group.

## Methods

### Research design and procedures

This study adopted an experimental approach to test the effectiveness of a human library intervention at increasing MHL. A multigroup pretest–posttest control group design was used. Such a design allows for a comparison of intervention effects between different intervention and control conditions [16]. Participants were randomly assigned to each of the following three groups: (1) the experimental group, in which the participants engaged in the human library intervention; (2) a comparison group, in which the participants engaged in a didactic educational session; and (3) a control group, in which the participants received no intervention. The interventions were conducted separately and transfer between groups was not allowed. Pretests and posttests were performed for all participants in all three groups to determine the level of MHL before and after the intervention session. The pretest was conducted before the day of intervention, while the posttest was performed one hour after the intervention program.

### Participants

Forty-five young adults were openly recruited from local tertiary educational institutions for participation in this study. Upon enrolment, participants were screened for eligibility using the following inclusion criteria: (1) they were young adults attending a local tertiary educational institution, and (2) they were mentally stable and able to comprehend the instructions in the assessment and training. Participants were excluded if they, or their close relatives, had been diagnosed with a mental illness. Cohen defined an effect size of more than 0.5 is considered to be clinically significant [17]. Our design provided adequate statistical power, with a sample size of 45. Using G*Power 3.0.10, an actual power of 0.9 was calculated, with an effect size of 0.6. The effect size was medium to large [17], with the level of significance set at 0.05.

Ethical approval was granted by the Human Research Ethics Committee of the University. Informed consent was obtained from all participants before their participation in the study. No remuneration was provided for participants in this study.

### Outcome measures

A modified version of the vignette-based MHL scale [18] was used in this study. The scale was adapted based on recent relevant evidence [10, 18, 19] and comprised three MHL subscales: (1) knowledge, which included a recognition of mental disorders and knowledge of help-seeking options [18]; (2) perceived stigma [10]; and (3) discrimination, which included preferred social distance [19]. There were 51 items in the vignette-based MHL scale, with 12 items in the knowledge domain, 24 items in the stigma domain, and 15 items in the discrimination domain (Table 1). Three sets of vignettes describing the major types of common mental health conditions (i.e. schizophrenia, affective disorder, and generalised anxiety disorder) were used in the questionnaire. Each set comprised two vignettes of the same mental disorder, one set was use in the pretest while the other set was used in the posttest. A vignette is a case scenario that describes the situation of an individual with a mental disorder. It is commonly used in testing MHL [2]. A high total score denotes a high MHL and vice versa. The reliability of the overall scale and the three subscales for the current sample set was confirmed (Table 1). Cronbach’s alpha of the overall scale in pretest was 0.81 and posttest was 0.90, which indicated a good internal consistency [16].

**Table 1:** The modified vignette-based MHL scale

	No. of items	Format	Max. score	Cronbach’s alpha	
				pretest	posttest
Overall MHL	51		210	0.81	0.90
Knowledge	12	Short quiz	30	0.78	0.74
Stigma	24	Multiple choice questions (5 options)	120	0.81	0.87
Social distance	15	Multiple choice questions (4 options)	60	0.84	0.89

### Interventions

The human library intervention was a one-off activity. The programme lasted for 2 hours and took place at a student development centre. There were two human books (people who shared their stories): a woman with schizophrenia and a man with bipolar disorder. At the beginning, a trained instructor introduced the rules for the activity, such as mutual respect, confidentiality, and genuineness. Readers had to be respectful in their questions and conversation with the Human Book and other Readers. Fifteen participants were then divided into two groups to take turns to read the books. Each group went to a room that had a comfortable setting, where everyone could sit freely and feel relaxed. This provided a safe space for communication with minimal pressure. Readers were briefed with the guidelines in attending the human library. When reading, the books first shared their story, including details on how they were diagnosed, their rehabilitation experience in hospital, how they see their illness, how they were supported, and the obstacles encountered when re-entering society. After the books finished sharing, the participants were free to ask questions. The questions were about how to support people with mental disorders and how to help reduce public stigma. At the end of the reading session, the participants wrote feedback for the books, such as messages of appreciation and encouragement.

The didactic educational programme involved a one-off teaching session that lasted 2 hours. In the session, a trained instructor used a comprehensive PowerPoint slideshow to teach the participants about common mental disorders. The session covered a basic understanding of mental illness, including its aetiology, signs and symptoms, common interventions, and available services. Recent statistics and mental health needs of the local community were also included in the training session to enable a comprehensive understanding of common mental disorders.

For participants in the control group, there was no intervention provided throughout the course of the programme.

### Data analysis

Descriptive statistics were used to illustrate the profile of MHL in the three groups according to the sub-domains and the three mental health conditions. To test the effectiveness of the human library intervention at improving MHL, a one-way analysis of covariance (ANCOVA) was used to compare the dependent variable, which was the overall MHL, in the three groups with the pretest score of the control group. To test the effect of the human library intervention on knowledge, perceived stigma, and social distance, a one-way multivariate analysis of variance (MANOVA) was used to account for the relationships among the three dependent variables (knowledge, stigma, and social distance) when comparing the three groups. To test the effect of the human library intervention on overall MHL, a MANOVA was used to determine if there were any differences between the three groups in the overall MHL related to schizophrenia, depression, and anxiety disorder in both the pretest and posttest.

## Results

The study included 45 participants, of which 14 were male and 31 were female. Their ages ranged from 19 to 23 years (mean = 21.3, standard deviation = 0.65). All participants were attending tertiary institutions. Descriptive statistics showing the profile of the three groups are provided in Table 2.

**Table 2:** Descriptive statistics for the MHL scores in the three groups

	Human library		Didactic teaching		No intervention	
	Pretest	Posttest	Pretest	Posttest	Pretest	Posttest
**Domains**						
Knowledge score	9.87 (4.98)	15.93 (4.28)	8.20 (4.75)	14.07 (3.20)	9.53 (4.34)	13.33 (5.6)
Stigma score	75.60 (11.27)	81.47 (11.36)	77.40 (9.77)	73.73 (13.20)	71.20 (8.49)	71.67 (8.03)
Social distance score	37.00 (6.52)	44.60 (6.57)	38.80 (4.59)	35.60 (4.27)	37.33 (4.91)	36.47 (4.09)
**Mental health conditions**						
Schizophrenia	40.67 (4.84)	47.87 (6.31)	40.80 (6.69)	42.13 (5.53)	38.40 (6.08)	42.00 (5.11)
Depression	40.07 (4.54)	45 ( 4.05)	41.07 (5.56)	38 (6.58)	39.27 (6.37)	38.13 (5.25)
Anxiety disorder	41.73 (5.89)	49.13 (7.98)	42.53 (5.54)	43.27 (7.62)	40.4 (3.81)	41.33 (3.94)
**Overall MHL score**	122.47 (13.08)	142.00 (16.41)	124.40 (12.81)	123.40 (17.78)	118.07 (13.66)	121.47 (11.61)

### Intervention effects – overall MHL

The primary outcome measure was overall MHL. A one-way ANCOVA was performed to compare the effectiveness of the three interventions whilst controlling for the pretest score. Levene’s test and normality checks were carried out, and the assumptions were met. There was a significant difference in the posttest overall MHL score (F [2.41] = 9.263, *p* = 0.000, effect size *d* = 1.34) between the groups. Post hoc tests showed a significant difference between the experimental and comparison groups (*p* = 0.001), and between the experimental and control groups (*p* = 0.003). However, there was no significant difference between the comparison and control groups (*p* = 1.00). On comparing the estimated marginal means, the highest MHL was found in the experimental group (M = 141.56) compared with the comparison (M = 121.91) and control groups (M = 123.41).

### Intervention effects – knowledge, stigma, and social distance

The three subscales reflected three essential aspects of MHL, namely, knowledge, stigma, and social distance. A one-way MANOVA was used to determine whether there were any differences between the three groups in term of the three subscale scores in the pretest and posttest. The MANOVA indicated no significant differences between the groups for knowledge, stigma, or social distance scores (F [6, 80] = 0.73, *p* = 0.631) in the pretest. In the MANOVA for posttest scores, there was a statistically significant difference in subscale scores between the three groups (F [6, 80] = 4.16, *p* = 0.001; Wilk’s Λ = 0.58; partial η^2^ = 0.24). Significant differences were found in the stigma subscale score (F [2, 42] = 3.27, *p* = 0.04, effect size d = 0.79) and the social distance subscale score (F [2, 42] = 14.21, *p* = 0.000, effect size d = 1.64). However, no significant difference was found in the knowledge subscale score (F [2, 42] = 1.349, *p* = 0.27). Post hoc univariate tests revealed the source of the significant main effects. Significant differences were found in the social distance score between the experimental group and both the control and comparison groups (*p* < 0.01); and in the stigma score between the experimental and control groups (*p* < 0.05).

### Intervention effects – MHL for schizophrenia, depression, and anxiety disorder

Three sets of vignettes measured the participants’ MHL for schizophrenia, depression, and anxiety disorder. A MANOVA was used to determine if there were any differences between the three groups in the MHL for the three mental health conditions in the pretest and posttest. No significance differences were found between the groups for MHL scores for schizophrenia, depression, or anxiety disorder in the pretest (F [6, 80] = 0.34, *p* = 0.91). In the MANOVA of the posttest scores, there was a statistically significant difference in the dependent variables (namely, schizophrenia, depression, and anxiety disorder) between the three groups (F [6, 80] = 2.85, *p* = 0.01; Wilk’s Λ = 0.68; partial η^2^ = 0.18). Significant differences were found in the MHL for schizophrenia (F [2, 42] = 5.23, *p* = 0.009, effect size d = 1.00), depression (F [2, 42] = 8.27, *p* = 0.001, effect size d = 1.24), and anxiety disorder (F [2, 42] = 5.41, *p* = 0.008, effect size d = 1.04). Post hoc univariate tests revealed the source of significant main effects. Significant differences in MHL for schizophrenia (*p* < 0.05) and depression (*p* < 0.01) were found between the experimental group and both the control and comparison groups. For MHL regarding anxiety disorder, a significant difference was only found between the experimental and control groups (*p* < 0.01).

## Discussion

This study demonstrated that the human library intervention significantly improved overall MHL, as compared with didactic teaching (comparison group) or no intervention (control group). In the human library intervention, the participants were able to immediately ask questions to clarify their misunderstandings, and thus, they gained a better understanding of the person (human book) and their disabilities. Face-to-face encounters of the participants with people with mental illness literally narrowed the social distance by placing them together in a real-life situation. Contact is essential to decrease prejudice. Intergroup contact theory suggests that contact, under certain optimal conditions, decreases prejudice and discrimination [20]. A human library provides an opportunity for intergroup contact under such optimal conditions, as there is equal status between the person with the mental illness and the audience; common goals of enhancing communication and reducing prejudice are shared during the session; and intergroup cooperation is encouraged. According to Fisher’s narrative paradigm theory, all meaningful human communication can be viewed as narration and can be effective when people see good reasons for adopting the point of view advocated in the communication [21]. The lived experience, as communicated through the human library session, may change people’s attitudes because the stories and experiences shared are all true and authentic. This promotes respect, acceptance, and mutual understanding towards both the person and the disabilities.

MHL in this study was conceptualised as a construct comprised of three domains: knowledge of mental illness, stigmatisation towards mental illness, and the maintenance of a preferred social distance from people with mental illness. The MANOVA results showed that the human library was effective at reducing stigma and social distance, but did not affect knowledge acquisition. This study confirmed previous data showing that human library intervention promotes inclusion by reducing stigma and social distance [14, 15]. However, the results of this study showed that participants in neither of the three groups demonstrated a significant improvement in the acquisition of knowledge about mental health. This result may imply that a single, stand-alone mental health information session, or even a human library session, is not sufficient to promote a significant intellectual change. To enhance effectiveness, information and awareness-raising sessions may need to be incorporated in a curriculum, rather than conducting them as one-off sessions, and the consumer-educator (i.e. the person with mental health issues) may need to be involved in the training activities [11].

This study demonstrated that the didactic teaching approach was not effective at enhancing MHL. MHL involves more than health education [22]. The didactic teaching approach (comparison group) had limited effects because it may have overloaded the participants with information. Because of the limitations and restrictions in processing capacity in such a relatively brief training session, participants in a 1-hour-long session can only be presented with a limited amount of information, and even less of this information is then encoded and learnt [23]. Moreover, solely providing knowledge, without contact, is not sufficient to improve the essential components of MHL, such as help seeking and reducing stigma and discrimination [20]. These findings echoed those of a previous study, in which lecture-based teaching on MHL did not significantly improve attitudes (stigma) or behaviours (discrimination) towards mental illness [24].

This study also assessed the level of MHL among the three groups using three sets of vignettes to illustrate the three mental health conditions (schizophrenia, depression, and anxiety disorder). The human library intervention was found to be significantly effective at increasing MHL for schizophrenia, depression, and anxiety disorder. It is worth noting that in the human library session, two guests (human books) were included: one with schizophrenia and the other with depression. As such, anxiety disorder was not formally introduced in the human library session. The improvement in the MHL scores in all three conditions may imply that the human library did have a generalised effect on reducing stigma and social distance and increasing the understanding of help seeking.

MHL is an evolving construct that includes an understanding of mental disorders and help-seeking options (knowledge) and decreasing stigma (attitudes) and social distance (behaviour). To enhance the effectiveness of the human library intervention at improving MHL, all three components (knowledge, attitudes, and behaviours) of MHL should be addressed in a balanced manner. There is currently no standardised protocol for human library intervention. It is not feasible or practical to have a standardised protocol for a human library, because it emphasises the sharing of unique and personal experiences. However, it is recommended that the planning of the human library session should follow a gross framework to include all three components: knowledge, attitudes, and behaviours. To enhance knowledge acquisition, it may be necessary to include some educational materials to enable the participants to learn and understand the specific types of disabilities. To address the issues of stigma and discrimination, it is necessary to focus on the impact of the disability on activities and social participation in relation to social and cultural contexts. The adoption of a community-based inclusive development model is preferred rather than a medical-oriented and impairment-based model. Unlike the medical approach, which has a narrow focus on the signs and symptoms of an illness, a community-based inclusive development model emphasises the rights of people with disabilities to participate equally in the community [25].

Strengths and limitations of this study were explored. This study contributes to the evidence-based practice of human library and MHL interventions. It is pioneer research in Chinese communities examining the impact of a human library intervention on participants. It followed the recent trends and latest conceptualisations of MHL by including the domains of acquisition of mental health knowledge, stigma, and preferred social distance in the process. A homogeneous group of participants (i.e. young adults attending tertiary education institutes) was targeted, and this enabled a valid comparison between the experimental, comparison, and control groups. This study has some limitations. This study involved more females than males and it might affect the generalizability of study results. Although a power analysis demonstrated that the sample size of this study (*n* = 45) achieved sufficient power, its validity may have been enhanced if the sample size was increased. This study adapted the MHL scale as an outcome measure. Further development and testing of this adapted scale are warranted to enhance its validity and reliability. Moreover, the concept of MHL and the validity of the construct are evolving and should be further confirmed and clarified to ensure the validity of the various testing methods.

Future research development into this area is indicated. To further test the efficacy of the intervention, it may also be necessary to examine the maintenance of the intervention effect after terminating the intervention. Follow-up assessments to test for the sustained effectiveness of stigma reduction intervention is deemed essential [26]. Moreover, a qualitative approach can be adopted to enhance the understanding of human library intervention. A quantitative approach could capture mainly the quantitative change of the participants upon the intervention. Important qualitative change of the participants and the human library process itself was not captured in this study. It may be interested to look into the interaction between the participants (readers) and the human books in using a qualitative approach. Using a qualitative approach, it will be able to capture also the qualitative change in awareness, thoughts and behaviours as a result of the intervention to improve MHL. Such qualitative outcomes might include a change in interaction pattern with the people with mental health issues, increased altruistic behaviour towards those in need or made donations to mental health charities.

## Conclusions

This study confirmed the effectiveness of a human library intervention at improving MHL. The human library intervention specifically reduced the stigma towards and perceived social distance from people with mental illness. As an innovative form of intervention to enhance MHL, it is suggested that the planning of the programme adhere to the overall goals of improving the acquisition of knowledge about mental illness and reducing stigma and social distance. Further studies are recommended to further develop the content of the human library intervention and to validate the constructs and related measurements of MHL.

## Data Availability

The datasets used and/or analysed during the current study available from the corresponding author on reasonable request.

## Abbreviations

MHL: Mental health literacy
LGBT: Lesbian, gay, bisexual, and transgender
ANCOVA: A one-way analysis of covariance
MANOVA: Multivariate analysis of variance
